# How to inhibit breast cancer and breast cancer metastasis with Akt inhibitors: Lessons learned from studies in mice

**Published:** 2021

**Authors:** Nissim Hay

**Affiliations:** 1 Department of Biochemistry and Molecular Genetics, College of Medicine, University of Illinois at Chicago, Chicago, IL 60607, USA; 2 Research & Development Section, Jesse Brown VA Medical Center, Chicago, IL 60612, USA

## Abstract

The PI3K/Akt signaling pathway is frequently hyperactivated in different types of breast cancer. In the past two decades, major efforts have been made to develop inhibitors of this pathway to treat cancer patients. However, the most evolutionarily conserved function of this pathway is in cellular and organismal metabolism, which is hijacked by cancer cells. Thus, adverse metabolic consequences are expected when PI3K or Akt is targeted. These metabolic consequences, particularly hyperinsulinemia, could impede the efficacy of treatment. This review summarizes recent genetic studies in mice that could pave the way to efficient breast cancer and breast cancer metastasis treatment with Akt inhibitors.

The serine/threonine kinase Akt is activated by extracellular signals that activate PI3K, such as tyrosine kinase growth factor receptors. Activation of PI3K generates PIP3, which binds to the pleckstrin homology (PH) domain of Akt and promotes an allosteric change in Akt and subsequent membrane binding and phosphorylation for full activation by PDK1, which is itself activated by PIP3, and by mTORC2. PI3K and Akt are negatively regulated by PTEN, which is a phospholipid phosphatase that dephosphorylates PIP3 generated by PI3K. Hyperactivation of Akt frequently occurs in human cancers as a result of loss-of-function mutations in PTEN, activation mutations in the genes encoding the catalytic subunits of PI3K, activation mutations in the PH domain of Akt, gene amplification of the Akt and PI3K genes, Ras activation, and activation of growth factor receptors (Figure 1).

For more than two decades, PI3K and its downstream effector Akt have been considered therapeutic targets for cancer. It took several years for pharmaceutical companies to develop specific inhibitors of the PI3K/Akt signaling pathway, some of which have recently been approved for the treatment of cancer. However, both PI3K and Akt have different isoforms encoded by different genes (Figure 1), and many of the developed drugs are pan-inhibitors that inhibit all isoforms, especially those that inhibit Akt. Patients who were treated with these inhibitors exhibited side effects that either curbed the efficacy of the drugs or prohibited further treatment [1]. A major side effect that can curb the efficacy of these inhibitors is the induced hyperinsulinemia [1]. The high circulating insulin level, at least in some cancers, further activates PI3K/Akt signaling and other pro-tumorigenic signaling pathways. The observed hyperinsulinemia is largely a consequence of Akt2 inhibition, which is the major Akt isoform expressed in insulin-responsive tissues and particularly in the liver. Akt2 deficiency in mice was documented to induce hyperinsulinemia [2,3], which precludes inhibition of tumorigenesis [4,5], and if partial deficiency of Akt1 also occurred, the mice developed hyperglycemia and diabetes [6]. This is also true in humans with a germline mutation in Akt2 that causes diabetes, in which the mutation which inhibits Akt2 activity, and the resulting mutant perhaps acts as dominant negative over the other Akt isoforms [7]. Thus, Akt2 inhibition should be avoided when designing drugs that inhibit Akt for cancer therapy.

Akt is hyperactivated in breast cancer by activation mutations in the catalytic subunit of PI3K, PTEN loss-of-function mutations, and Akt1 activation mutations. Activation mutations in *PIK3CA,* the gene encoding the catalytic subunit of PI3K, p110a, occur in 48% of invasive lobular carcinoma [8] and in 41%, 40%, 31%, and 14% of hormone-positive HER2-negative (HR+HER2), HR+HER2+, HR-HER2+, and triple-negative breast cancer (TNBC) breast cancer, respectively [9]. PTEN inactivation mutations occur in 9%, 7%, 2%, and 15% of HR+HER2-, HR+HER2+, HR-HER2+, and TNBC breast cancer, respectively, and Akt1 activation mutations occur in 7%, 2%, and 3% of HR+HER2-, HR+HER+, and TNBC breast cancer, respectively [9]. In addition, in HER2- or ERBB2-enriched breast cancer, dimerization with HER3/ERBB3 elicits robust activation of PI3K and Akt, since HER3/ERBB3 possesses six docking sites for PI3K (Figure 1).

The roles of the different Akt isoforms in breast cancer tumorigenesis and metastasis are complex and controversial [5]. To explore the therapeutic effect of the two out of three Akt isoforms (Akt1 and Akt2) in breast cancer and breast cancer metastasis, we employed mouse models that recapitulate HER2-enriched breast cancer and luminal B breast cancer. To emulate drug therapy, we systemically deleted Akt1 or Akt2 after tumor onset [5]. In both mouse models, systemic Akt2 deletion elevated insulin levels and either did not affect tumor progression or exacerbated tumor growth and metastasis. This is in contrast to the cell autonomous deletion of Akt2, which inhibited tumor growth. Thus, the results showed that the inability of Akt2 inhibition to affect tumor growth and metastasis, and even to exacerbate them, is due to its systemic effect. Indeed, treatment with the antidiabetic drug sodium-glucose cotransporter (SGLT2) inhibitor, canagliflozin, decreased insulin levels after systemic Akt2 deletion and increased the efficacy of systemic Akt2 deletion. Interestingly, treatment with the antidiabetic drug metformin did not have a significant effect on hyperinsulinemia induced by Akt2 deficiency. Since canagliflozin was shown to inhibit hyperglycemia and hyperinsulinemia without adverse consequences [10], cotreatment with Akt or PI3K inhibitors that inhibit Akt2 in insulin-responsive tissues should be considered. Interestingly, the diabetic drug metformin did not have any significant effect on hyperinsulinemia induced by systemic Akt2 deletion in mice [5].

An inhibitor of PIK3CA, alpelisib, has been approved for the treatment of hormone-positive HER2-negative (HR+HER2-) breast cancer, in which *PIK3CA,* the gene encoding p110a, is frequently mutated. However, since *PIK3CA* is ubiquitously expressed in diverse types of tissues, including insulin-responsive tissues, the use of this inhibitor could elicit hyperinsulinemia and diabetes. Indeed, it has been reported that breast cancer patients who receive alpelisib can develop hyperglycemia and diabetic ketoacidosis [11–13]. It was documented that, to increase the efficacy of p110a inhibitors in mice, cotreatment with a ketogenic diet or SGLT2 inhibitor is required [14]. However, the reported diabetic ketoacidosis as a consequence of treatment with the PIK3CA inhibitor could raise concerns about the application of ketogenic diet.

When Akt1 was systemically deleted, it not only inhibited tumor growth but also markedly inhibited metastasis. This is in contrast with the cell autonomous deletion of Akt1, which did not inhibit metastasis [5]. Once again, there is a discrepancy between the cell-autonomous inhibition of Akt1 and its systemic inhibition. Since drug therapy is systemic, we should pay close attention to the systemic deletion of the Akt isoform. Moreover, the systemic deletion of Akt1 inhibited the metastasis breast cancer tumors even though Akt1 expression was intact [5]. These results clearly demonstrate that the effect of Akt1 inhibition on breast cancer metastasis is largely systemic. Sigle cell RNA sequencing of the primary tumors showed several tumor specific clusters, which were intact after Akt1 systemic deletion. However, tumor associated neutrophils were not present after Akt1 systemic deletion, indicating that inhibition of Akt1 prevented prometastatic neutrophils from promoting metastasis. Indeed, the deletion of Akt1 only in neutrophils was sufficient to inhibit metastasis [5]. Neutrophils in animals bearing tumors are different than naive neutrophils [15]. They are reprogrammed in the bone marrow by cytokines, most notably G-CSF, secreted by tumor cells [15,16]. They are reprogrammed both metabolically and to express high levels of pro-angiogenic and pro-invasive factors such as *S100a8*, *S100a9*, *MMP8*, *MMP9*, *Bv8*/*Prok2* and vascular endothelial growth factor (*Vegfa).* Thus, tumor-associated neutrophils (TANs) could promote the migration, invasiveness, and extravasation of tumor cells (Figure 2A). Neutrophils play a prometastatic role in breast cancer [17,18], and a high neutrophil to lymphocyte ratio (NLR) is associated with worse overall survival and disease-free survival outcomes [19]. It has been shown that neutrophils cluster with circulating tumor cancer cells in patients with invasive breast cancer, escorting these circulating cells and promoting their proliferation and metastatic potential [20]. It should be noted that the role of neutrophils in breast cancer metastasis was documented mostly with metastasis to the lungs. However, metastatic breast cancer tumors also frequently metastasize to the bone as well as to the brain and liver [21]. Although it remains to be seen whether neutrophils also promote metastasis to these organs, the documented role of neutrophils in escorting circulating tumor cells to the metastatic site [20], suggest that they also promote metastasis to other organs.

The recognition of the role of neutrophils in promoting tumor growth and metastasis led to clinical trials (NCT03161431, NCT04477343, and NCT04599140) in cancer patients to evaluate inhibitors of CXCR2, which is the chemokine receptor that promotes neutrophils mobilization and migration. It is common practice to treat patients with G-CSF after chemotherapy with G-CSF; however, since G-GCF can also promote the generation of prometastatic neutrophils, this practice might need to be revisited.

The systemic deletion of Akt1 does not exert adverse consequences [5] and does not affect the number of peripheral neutrophils or other functions of neutrophils [5,22]. Therefore, the effect of systemic Akt1 deletion on prometastatic neutrophils but not on the other functions of neutrophils indicates that Akt1-specific inhibitors would be sufficient to selectively inhibit the prometastatic effect of neutrophils. Moreover, the results suggest that specific Akt1 inhibition inhibits breast cancer metastasis regardless of the origin of the primary tumor. Currently, there are no specific Akt1 inhibitors, but in principle, these could be developed. Indeed, an allosteric Akt1 selective inhibitor has been previously reported [23]. Alternatively, pan-Akt inhibitors that selectively avoid Akt2 inhibition could be employed (Figure 2B). It remains to be seen whether selective Akt1 inhibitors will be developed in the near future to treat breast cancer metastasis.

## Figures and Tables

**Figure 1:**
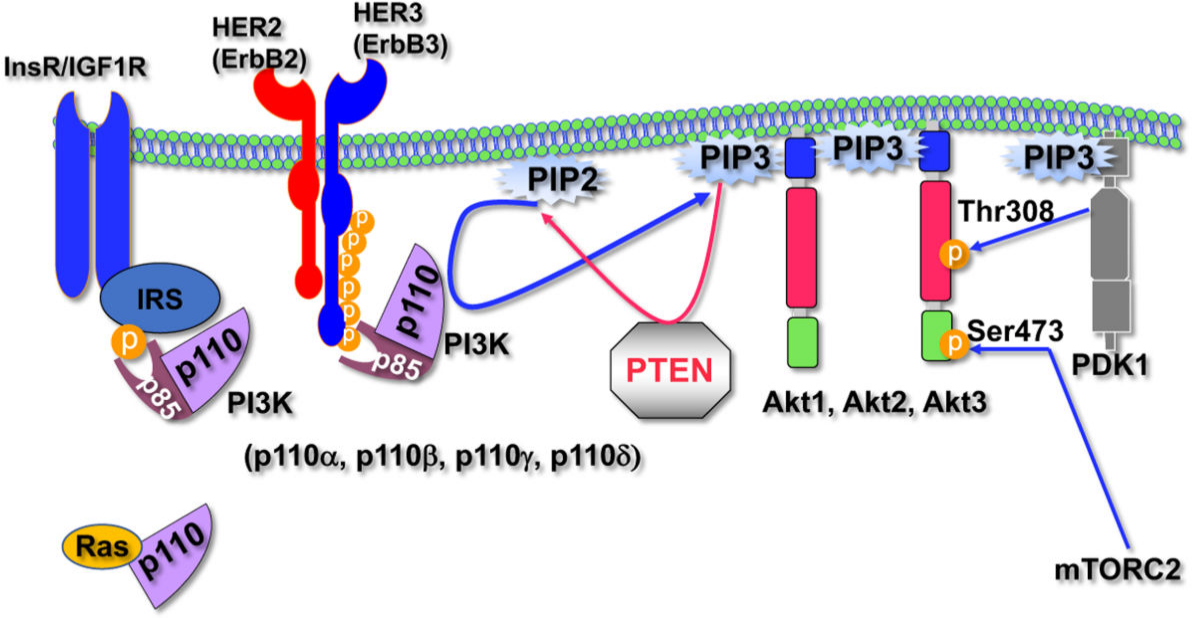
Activation of Akt by growth factor receptors. The insulin receptor or IGF1 receptor activates the catalytic subunit of PI3K, p110, by activating insulin receptor substrate (IRS), which in turn recruits the regulatory subunit of PI3K, p85. HER2 activates PI3K by heterodimerization with HER3, which possesses six docking sites for p85. Ras, which is also activated by growth factor receptors, can also activate p110 through physical interactions. Activated p110 generates PIP3 from PIP2. PIP3 binds the PH domain of Akt to target it to the plasma membrane and is fully activated by PDK1 and mTORC2, which phosphorylate Akt at different residues. PTEN counteracts p110 activity by dephosphorylating PIP3 back to PIP2. The different p110 and Akt isoforms are indicated.

**Figure 2:**
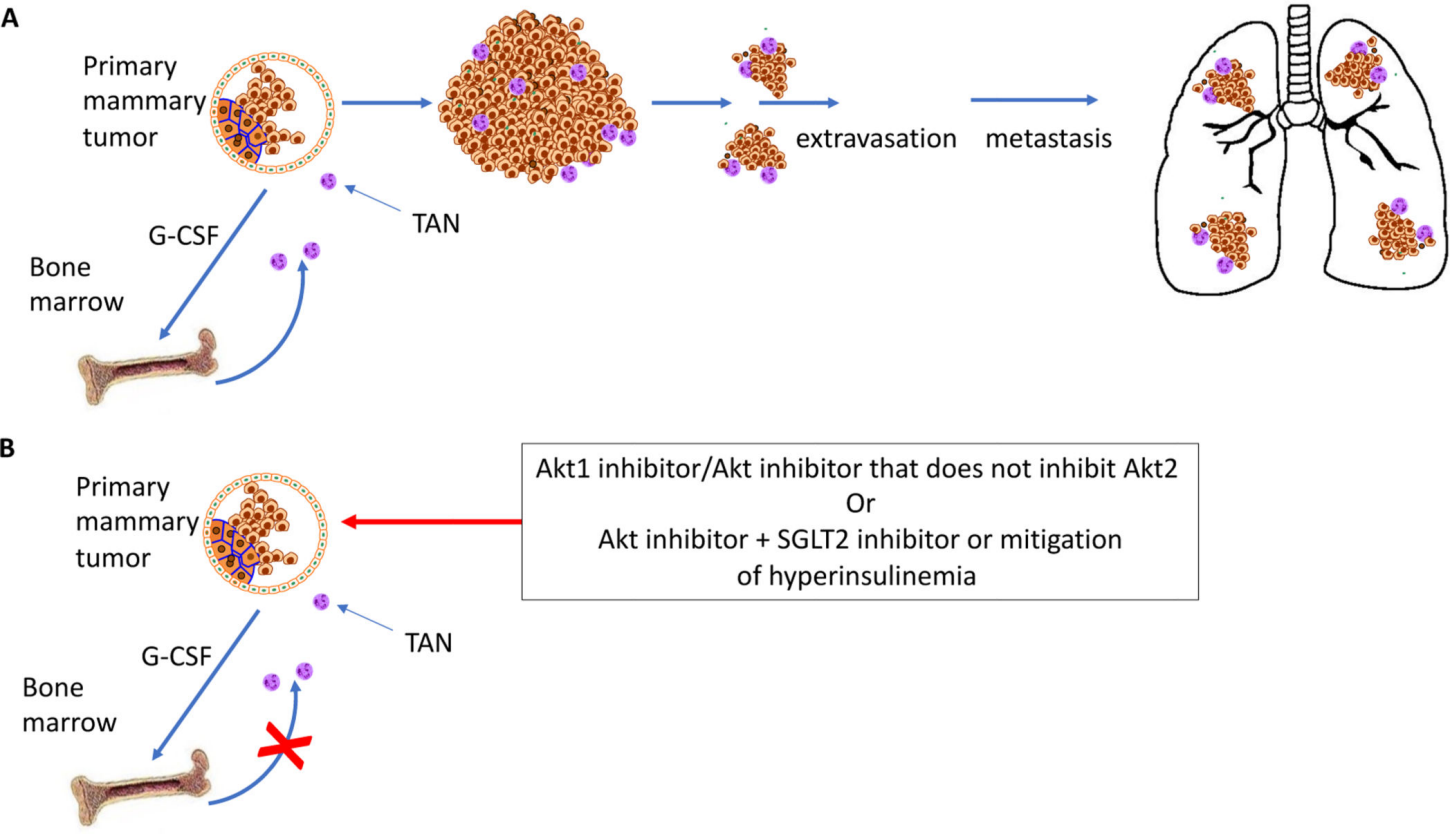
Tumor-associated neutrophils (TANs) promote breast cancer metastasis, which is inhibited by Akt1 inhibition. **A.** Primary mammary tumors secrete G-CSF and other cytokines to reprogram neutrophils in the bone marrow and facilitate their migration to the tumor site. Neutrophils then associate with circulating tumor cells and promote their extravasation and metastasis. **B.** Selective Akt1 inhibitors or Akt inhibitors that do not inhibit Akt2 inhibit breast cancer metastasis by impairing programmed neutrophil mobilization and survival.
